# Supplementary material for: The causal relationship between the human gut microbiota and pyogenic arthritis: a Mendelian randomization study
Source: Front Cell Infect Microbiol. 2024 Nov 26;14:1452480. doi: 10.3389/fcimb.2024.1452480 (PMC11629706; doi:10.3389/fcimb.2024.1452480)
Supplement: Supplementary file 1 [file DataSheet1.zip › mendelian test/ebi-a-GCST90027573.csv_leave_one_out.pdf]

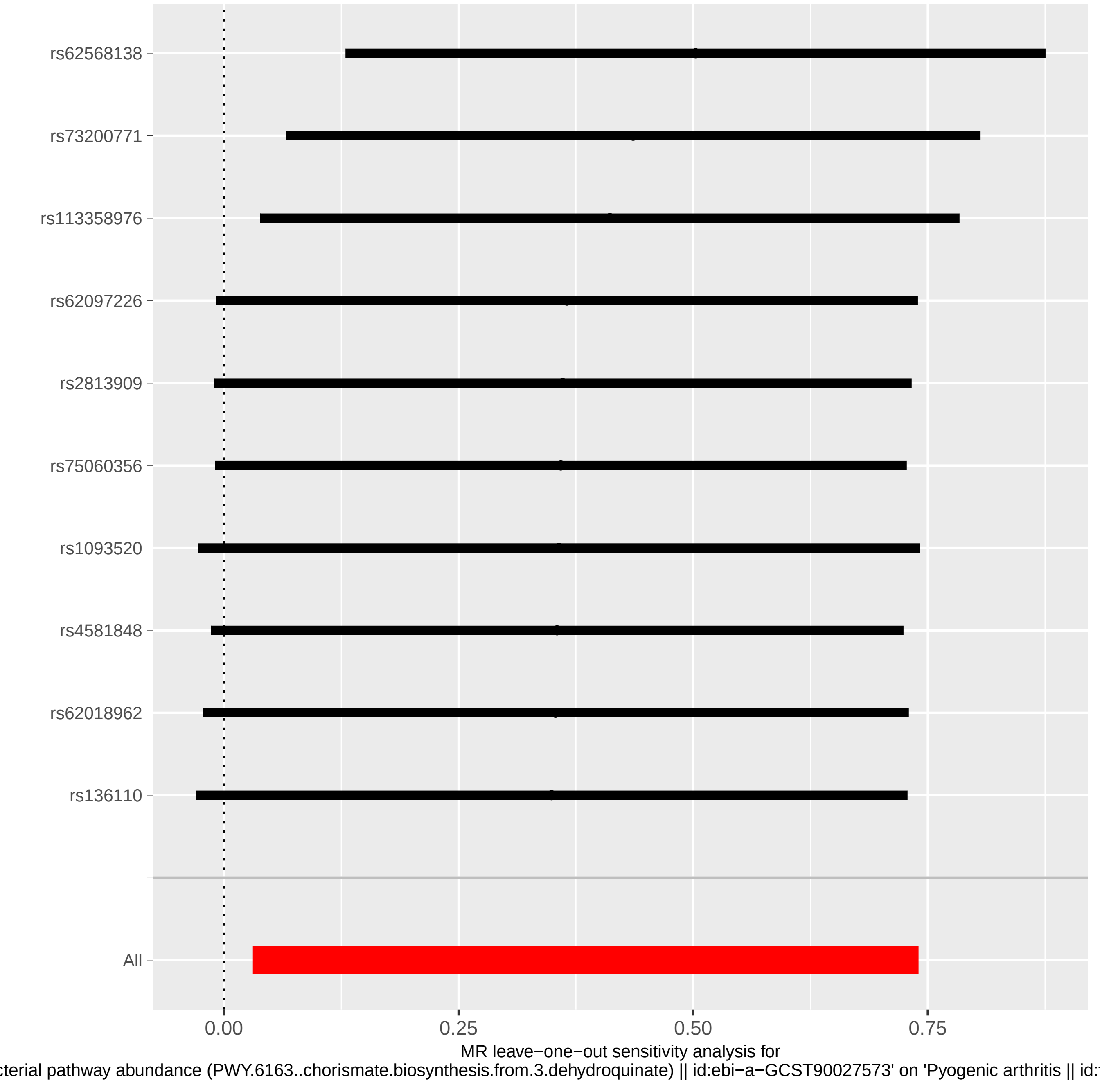
rs62568138
rs73200771
rs113358976
rs62097226
rs2813909
rs75060356
rs1093520
rs4581848
rs62018962
rs136110
All
0.00
0.25
0.50
0.75
MR leave−one−out sensitivity analysis for
cterial pathway abundance (PWY.6163..chorismate.biosynthesis.from.3.dehydroquinate) || id:ebi−a−GCST90027573' on 'Pyogenic arthritis || id:
